# The Bacterial Swiss Army Knife: ExPEC Utilizes Multiple Resistance Mechanisms to Counteract Host Immune Responses

**DOI:** 10.3390/vaccines14010051

**Published:** 2025-12-31

**Authors:** Eveline Weerdenburg, Susan King, Joyce Lübbers, Elise Hovingh, Todd Davies, Jeroen Geurtsen, Germie van den Dobbelsteen, Jan Poolman

**Affiliations:** 1 Janssen Vaccines & Prevention, Johnson & Johnson, 2333 CN Leiden, The Netherlands; 2 Janssen Research & Development, Johnson & Johnson, Raritan, NJ 08869, USA

**Keywords:** extraintestinal pathogenic *Escherichia coli*, serum resistance, complement system, polysaccharides, opsonophagocytosis

## Abstract

Extraintestinal pathogenic *Escherichia coli* (ExPEC) is a major cause of infections of the urinary tract, the bloodstream, and other non-intestinal sites in humans. ExPEC often resists the bactericidal action of human immune defenses including complement, antimicrobial peptides, antibodies, and cell-mediated killing. This review provides an overview of the main host defense strategies, and the mechanisms and molecules ExPEC engages to resist these human immune responses. Surface-exposed polysaccharides, outer membrane proteins, cytotoxins, and proteases are all part of the bacterial arsenal of defenses that can neutralize many of the host’s immune defenses. These factors work in concert to enable ExPEC to survive and thrive in extraintestinal environments of the human body.

## 1. Introduction

The Gram-negative *Escherichia coli* (*E. coli*) is a multi-faceted bacterial species. Its members may reside as part of the gut microbiota in the human gastrointestinal tract, but can also cause disease [1]. The human gut microbiota contains over 1000 species-level bacterial phylotypes, and *E. coli* is part of this mixture in almost all (>90%) humans [2,3]. In addition to this non-pathogenic *E. coli*, the species includes numerous disease-causing pathotypes that cause intestinal or extraintestinal disease (for a review of *E. coli* pathogroups with their clinical presentation and genetic markers, see [4]).

*E. coli* has evolved to survive in different anatomical locations through the acquisition of niche-specific combinations of pathoadaptive traits. By virtue of the many virulence factors expressed to allow survival in the host, the bacteria are potentially equipped to outmaneuver a plethora of host immune defenses, including both soluble and cell-mediated effector mechanisms. As a consequence, expression of a conducive combination of virulence factors can enable specific strains of *E. coli* to escape the immune response and cause symptomatic infections in niches outside the gastrointestinal environment. These strains are collectively known as extraintestinal pathogenic *E. coli* (ExPEC) [4]. The heterogeneous set of virulence factors that are characteristic for ExPEC strains allows them to inhabit a wide range of environments and withstand diverse host defense mechanisms. In contrast, intestinal pathogenic *E. coli* (InPEC) pathotypes are restricted to the gut where they cause diarrheal diseases using a limited but very effective set of virulence factors [4].

Already a century ago, the host response to bacterial infection was attributed to white blood cells and blood fluids [5]. The bactericidal effect of serum is mainly attributed to complement factors, but is also the result of the action of other soluble serum factors such as antimicrobial peptides (AMPs) or antibodies [6]. Serum resistance refers to the survival of bacteria in serum, observed in experiments to identify which bacterial species, strains, or mutants are susceptible or resistant to serum-mediated growth attenuation. A body of evidence has also accrued to show the importance of cell-mediated responses in controlling bacterial infection [7]. Clues to the key host and bacterial components at play come from genetic analysis of host and bacterial susceptibility, and mechanistic studies continue to shed light on the underlying molecular mechanisms behind these immune pathways and how these can be evaded by pathogenic bacteria including ExPEC.

This review outlines the main antibacterial host defense strategies used to prevent and control ExPEC infection and describes the function of ExPEC factors that act to disable these host defenses. First the ExPEC pathotypes and their extraintestinal niches are outlined, followed by evidence of major host defense mechanisms that act as the first line of protection from infection: complement activation, antimicrobial peptides, neutrophil-mediated responses, and antibody-mediated defenses. ExPEC expresses a multitude of bacterial factors that can counteract the host defenses, and these are described by their location as surface polysaccharides, outer membrane proteins, or secreted toxins and proteases. Attention is turned to how these virulence factors act in concert, for example, to impede complement activity, restrict access of host factors to the bacterial surface, and resist cell-mediated killing. Although phenotypes can differ between specific pathotypes, ExPEC strains generally express a broad array of virulence factors that allow for redundancy and reinforcement, together overpowering the immune response. Ultimately, it is the action of a combination of these factors that dictates the ability of an ExPEC strain to colonize and survive in extraintestinal sites and cause disease.

## 2. Extraintestinal Pathogenic *E. coli* Disease

ExPEC can enter normally sterile body sites, causing invasive *E. coli* disease (IED, also known as invasive ExPEC disease), in many different organ systems. In its most severe manifestation, IED can trigger sepsis, a life-threatening condition that happens when the body’s immune system responds too strongly to an infection, causing organ dysfunction and failure. ExPEC is also one of the main causes of neonatal infectious diseases and infectious stillbirths [8,9,10].

Uropathogenic *E. coli* (UPEC) forms the most prominent ExPEC pathotype, which is responsible for 70–95% of urinary tract infections (UTI) in the USA [11,12]. The source of UTI is usually *E. coli* transferred from the intestinal tract. The bacteria enter the urinary tract through the urethra, and, as they travel upstream along the tract, may cause cystitis (bladder infections) and prostatitis (infection of the prostate). Further upstream of the bladder, UPEC may travel along the ureters to the kidneys, which if left unchecked, results in pyelonephritis (infection of the kidneys). From any of these loci, the bacteria may proceed into the bloodstream, causing bacteremia (i.e., bloodstream infections) and, in severe outcomes, sepsis.

Although the urinary tract is a common source from which ExPEC can reach the bloodstream, the bacteria may also cross into the blood from the digestive tract. This frequently occurs before or after causing intra-abdominal infections (IAIs), including abscesses, peritonitis (infection of the tissue layer inside the abdomen), and single-organ infections such as diverticulitis, appendicitis, cholecystitis (infections of the gallbladder), cholangitis (infections of the bile ducts), or pancreatitis. Notably, *E. coli* is the most frequent Gram-negative bacillus found in these IAIs [13,14,15,16,17]. Furthermore, *E. coli* strains linked to chronic inflammatory bowel diseases such as ulcerative colitis display a close phylogenetic relationship to ExPEC [18,19,20] and may be able to cross into the bloodstream. In addition to the urinary and digestive tracts, ExPEC can enter the human body through other mucous tissue layers such as the airways, where it may cause ventilator-assisted pneumonia [21], and through the skin, where it can trigger skin and soft tissue infections [22].

ExPEC residing in the blood and causing clinical sepsis has been termed sepsis-associated *E. coli* (SEPEC) [12]. Notably, ExPEC accounts for 27% of all bacteremia cases worldwide [23], for more than 50% of all cases in Australia [24], and for about 37% of neonatal cases in the US [25]. Moreover, 34% of community-onset sepsis occurrences are caused by *E. coli* [26]. When transported around the human body in the blood, some bacteria may settle in the fluid and membranes surrounding the brain and spinal cords, causing meningitis. This pathology is largely restricted to infants, and the ExPEC pathotype responsible for these potentially fatal infections is called neonatal meningitis *E. coli* (NMEC). NMEC is responsible for 14–37% of acute bacterial meningitis cases in infants [27,28,29,30,31]. Residence of these bacteria in the mother’s rectovaginal flora could be a source for NMEC in the newborn, where contamination likely happens via the colonized birth canal.

ExPEC pathotypes are generally classified by their anatomical location and clinical signs of infection, and the potential of an ExPEC strain to cause specific disease correlates with the presence of specific virulence factors. As an example, the presence of the autotransporter toxin Vat, the yersiniabactin siderophore FyuA, the heme siderophore ChuA, and the fimbrial protein YfcV have been suggested as indicators of the capability of an ExPEC isolate to infect the urinary tract [32]. Although canonical genes delineating ExPEC pathotypes do not exist, most ExPEC strains that cause human infection belong to a small number of globally disseminated clones [33,34]. Consequently, while the entire species encompasses bacteria with 185 different O-antigens (components of the surface lipopolysaccharide), 53 different H-antigens (flagellar proteins) and 80 different K-antigens (capsular polysaccharides) [35,36,37,38,39,40], two thirds of current ExPEC strains found in human bloodstream infections contain one of only 10 different O-antigens [41,42]. Similarly, the most prominent capsule antigen, K1, is present in approximately 80% of NMEC *E. coli* strains [43,44], and in about 25% of ExPEC strains causing bacteremia [45]. These strains appear to contain a particularly successful combination of traits that enable them to cause disease in specific extraintestinal niches.

## 3. Main Host Defense Strategies Against Bacterial Infection

The host employs various soluble and cellular components of its innate and adaptive immune systems to neutralize ExPEC, including complement, antimicrobial peptides, neutrophils, and antibodies [11,46]. Over the last 15 years, several studies have sought to characterize the components of ExPEC resistance mechanisms at a genome-wide level, using transcriptomics and transposon mutagenesis approaches. These studies give insights into the immune-mediated pathways that need to be overcome by pathogenic *E. coli* to gain a survival advantage outside the gut. These immune responses are described in more detail in the following sections.

### 3.1. Complement

**Figure 1:**
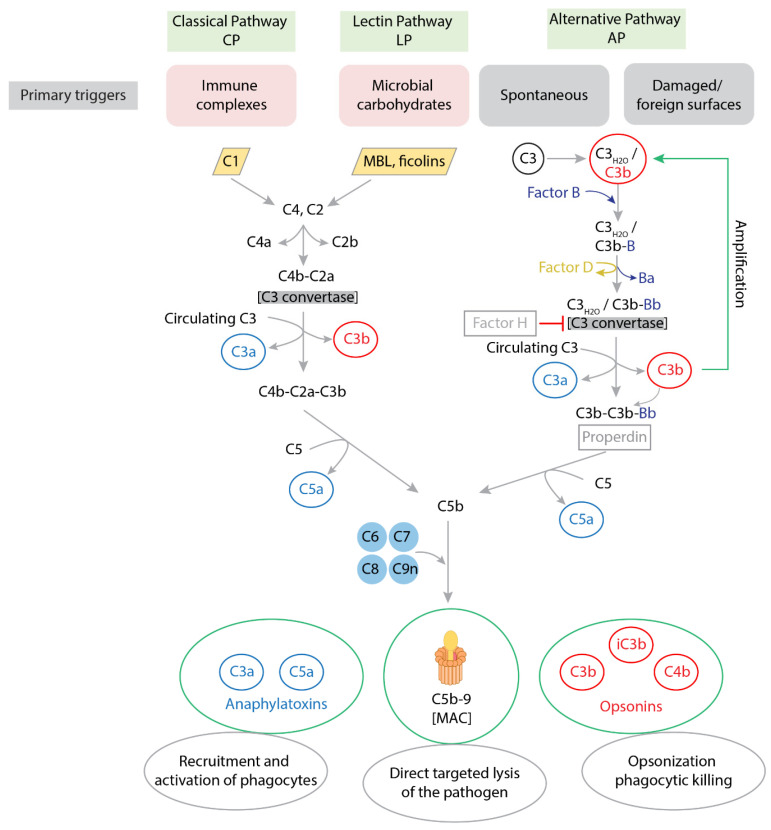
Overview of the three pathways of the complement system. All three pathways converge at forming a convertase enzyme that can convert complement component 3 (C3). In the first cascade-activating step of the classical pathway (CP) and the lectin pathway (LP), specific complement proteins either bind directly to the surface of pathogens or to pathogen-bound antibodies. The CP is primarily activated when C1 binds to bacterium-bound IgG or IgM [71]. In the LP, fluid-phase pattern-recognition molecules (PRMs) such as mannose-binding lectin (MBL) and ficolins recognize specific carbohydrate patterns on the pathogen’s surface. Both C1 and lectins recruit and convert C4 and C2, which results in the formation of a C3 convertase that converts C3 to C3a and functional C3b. The alternative pathway (AP) can be initiated by spontaneous hydrolysis of C3 into C3_H2O_, which similarly forms the soluble C3 convertase that generates C3b. The high density of C3b and association of molecules such as properdin allows the convertase enzyme to also convert the final complement component C5 into C5a and C5b; the latter forms the terminal membrane attack complex (MAC) with complement proteins C6–9.

When bacteria enter the human host, they are usually quickly recognized by pattern-recognition molecules (PRMs), antibodies, and complement proteins. Attachment of these molecules to the pathogen surface triggers activation of the complement system, which is present in blood plasma and tissues. This can occur via three main pathways, the classical pathway (CP), the alternative pathway (AP), and the lectin pathway (LP), as summarized in Figure 1 (reviewed in [47,48,49,50]). Once initiated, the complement cascade engages three major strategies to neutralize the bacteria. One strategy involves the release of proinflammatory mediators (anaphylatoxins) such as complement proteins C3a and C5a, leading to the recruitment and activation of phagocytes to deploy various antibacterial mechanisms. The second approach consists of opsonization, the marking of a pathogen-specific antigen for phagocytic removal. This can occur via IgG or IgM antibodies, or via components of the complement system such as C3b and its proteolytic fragments [51,52,53]. Phagocyte Fcγ receptors (FcγRs) and complement receptors (CRs) then recognize these pathogen-specific antibodies and complement components, respectively, and induce phagocytosis. Other types of receptors, such as C-type lectin receptors (CLRs), can bind directly to pathogen surfaces and promote phagocytosis [54]. The third complement-mediated strategy to neutralize bacteria is a direct targeted lysis of the pathogen via the terminal membrane attack complex (MAC) composed of complement proteins, which forms holes in the pathogen’s cell envelope that ultimately kill the microbial intruder [55,56,57,58].

The importance of the complement system for prevention of bacterial infections can be gleaned in hosts where this system is partially or entirely non-functional. Such defects can help pinpoint the exact immune pathways and components involved in an effective immune response against specific bacterial pathogens. However, such defects are rarely reported, and it has been suggested that less than 10% of CP and AP deficiencies and deficiencies of the late complement components are typically identified [59]. Overall, complement deficiency is detected in Western countries and Japan at a rate of about 0.03% of the population [60], although more recent frequency statistics show high variations around the world [61].

Sporadic case reports and investigations on small cohorts of patients with complement deficiencies showed a predisposition of affected patients to recurrent bacterial infections, bacteremia, and/or meningitis. Infections reported in individuals with complete deficiencies of CP components such as C1q/r/s, C4, and C2 include those with encapsulated bacteria such as *Streptococcus pneumoniae*, *Haemophilus influenzae*, and *Neisseria meningitidis* [60]. These bacterial organisms have also been found to cause invasive disease in the vast majority of individuals with deficiencies in complement factor I, the protein that sequentially cleaves C3b [62]. In fact, *N. meningitidis* infections in otherwise healthy young patients have been suggested to be indicative of complement deficiencies (usually, late complement component deficiency (LCCD), C5 to C9) [63]. The observed high incidence of invasive meningococcal disease in persons with LCCDs support the crucial role of MAC-dependent killing in battling *Neisseria* (reviewed in [64]). Recent data confirmed a strong association of LCCD with Neisserial infections while pneumococcal disease was restricted to patients exhibiting deficiencies in early CP components C1–C3 and factor I [65].

Remarkably, to date the incidence of *E. coli* infections has not been found to be increased in people with complement deficiencies. This could be due to a redundancy in mechanisms that protect from *E. coli* infection. Sporadic reviews mention *E. coli* among the pathogens frequently encountered in such patients [66,67], but cited sources lack specific evidence. The bacteria are frequently insensitive to complement, which could explain why they do not prominently emerge in subjects with complement deficiencies. Some of these complement-resistant *E. coli* strains exhibit little deposition of terminal MAC components and no damage to the cytoplasmic membrane [55,68]. Others show normal or increased complement activation and deposition of MAC components, but an incomplete assembly of the MAC, and release of soluble MAC from the bacterial surface [69,70]. The inefficiency of MAC-dependent killing of some *E. coli* strains suggests that (opsono)phagocytosis may overall constitute a more critical human defense mechanism against these bacteria. However, the concerted efforts of other first-line host resistance mechanisms (including AMP-derived protection and proinflammatory mediators of the early CP) may also contribute to prevention of ExPEC disease in immunocompetent human hosts.

### 3.2. Antimicrobial Peptides

In addition to complement factors, the innate immune system has a repertoire of AMPs (also known as human defense proteins, HDPs) which include a variety of small, usually cationic, peptides that exert bacteriolytic activity. AMPs are secreted by a variety of cell types, such as epithelial cells and neutrophils. They can be expressed constitutively or in response to infection. AMPs have a variety of functions beyond their direct antimicrobial activity, which include immunomodulatory properties, apoptosis, and wound healing [72,73]. Perhaps the most important human AMPs present in serum are defensins (α- and β-defensins) and cathelicidins, which act to disrupt bacterial membranes, inhibit cell wall synthesis and neutralize secreted toxins [74,75,76]. Deficiencies in AMPs lead to increased susceptibility to bacterial infections in humans. However, these deficiencies are not specifically linked to the development of ExPEC infections.

### 3.3. Neutrophils

Neutrophils form the main cellular innate defense following *E. coli* infection. A local release of chemokines leads to recruitment of neutrophils from the blood into the infected tissue, followed by activation of these cells at the site of infection. Neutrophils act to contain the infection by several mechanisms, including phagocytosis, degranulation, and neutrophil extracellular traps (NETs). Phagocytosis involves receptor-specific engulfment and intracellular lysis of bacteria that have been opsonized by soluble factors such as complement or antibodies, termed opsonophagocytosis. Bacteria can also be killed extracellularly by the release of antibacterial effector molecules such as neutrophil elastase, oxygen radicals, and AMPs in a process called degranulation [77]. In addition, neutrophils form NETs which create an extracellular matrix with a high concentration of AMPs at the site of infection.

The important role of neutrophils in bacterial defense is highlighted in individuals with neutrophil deficiencies. People with functional neutrophil problems such as chronic granulomatous disease and leukocyte adhesion deficiency are more susceptible to bacterial infections [78]. Of note, *E. coli* caused approximately 20% of bacterial infections in a group of 143 patients with ELANE (elastase produced by neutrophils)-related neutrophil deficiency, mostly in patients with severe chronic neutropenia, second only to *Staphylococcus aureus* [79]. Moreover, in neutropenic cancer patients undergoing antibiotic treatment, resistant *E. coli* has been described in several studies to cause a significant increase in bloodstream infections [80,81,82,83,84].

Neutrophil deficiency is especially devastating in neonates. Neonatal neutrophils differ profoundly from those in adults—they exhibit a fluctuating number of cell surface receptors such as CR3 and FcγIII [85] and reduced concentrations of microbicidal proteins and enzymes such as the bactericidal permeability-increasing protein BPI, impacting their ability to fight pathogens [86,87]. In addition, the neutrophil cell mass per gram body weight in neonates is only 25% of adult levels [88]. Perhaps this underdevelopment of the neutrophil component of the human immune system very early in life is an important contributor to the fact that *E. coli* is the main bacterial pathogen causing early onset neonatal sepsis in pre-term babies [89]. Interestingly, older adults might be vulnerable in a similar way. Although neutrophil levels in their blood and neutrophil precursors in their bone marrow are not reduced, as people age, the neutrophils’ ability to kill bacteria and their phagocytic index decrease. In neutrophils of older adults, production of reactive oxygen species and formation of NETs are impaired [90,91,92,93], and the cells’ chemotaxis capacity is reduced under multiple conditions [94]. Given the predominant resistance of *E. coli* against complement-mediated lysis and the resultant assumption of a more important role of opsonophagocytic killing mechanisms for these bacteria, reduced neutrophil functionality in people of advanced age might be an important factor related to the increased incidence of ExPEC infections in this population. The near absence of bacteremia in young adults and gradual age-dependent increase suggests that the decline in neutrophil function plays an important role, as has been suggested for other bacterial pathogens known to affect the elderly such as *S. pneumoniae* [95].

### 3.4. Antibodies

Antibodies are produced by B cells in response to infection, and are directed to specific protein or polysaccharide sequences, called epitopes, presented by the pathogen. Any subsequent infection by the same pathogen leads to a more rapid and substantial secretion of antibodies in a recall response from memory B cells. Antibodies play an important multifaceted pathogen-specific role in immune defense and control bacterial infection in several ways [96]. Antibodies can inhibit bacterial adherence to host cells through neutralization, i.e., hindrance of bacterial-host cell binding and entry. In addition, direct binding of antibodies to bacteria can initiate several bactericidal mechanisms, including opsonophagocytosis, antibody-dependent cellular cytotoxicity, and complement activation. Antibody isotypes IgM and IgG are potent activators of these bactericidal processes [52,97,98]. IgM is produced first in response to new infections, and this response is enhanced by the subsequent development of IgG, which is also rapidly produced in recall responses [99]. The secretory antibody isotype IgA (sIgA) is a dimeric antibody present at mucosal surfaces and plays a role in protection from infection in the respiratory and urogenital systems through multiple mechanisms. sIgA has oligosaccharide chains which bind to the fimbria of *E. coli* and inhibit bacterial adhesion [100,101], and is also resistant to host and microbial enzymes. sIgA can activate the lectin and alternative pathways for complement activation [102].

The extensive antigenic variability of surface-exposed structures on ExPEC points to the evolutionary need to avoid antibody recognition, allowing survival of the species, which underlines the importance of antibody-mediated protective mechanisms for the host. Despite the role of antibodies in the clearance of bacteria, and the susceptibility of individuals with severe combined immunodeficiency (SCID) or common variable immune deficiency (CVID) to bacterial infections in general, there is no widespread reporting of a link between SCID or CVID and susceptibility to ExPEC infections in particular [103,104,105].

## 4. Major ExPEC Factors Thwarting the Host’s First-Line Immune Responses

ExPEC resists the major host defense mechanisms with a multitude of factors, some of which serve to protect against more than one of the antibacterial immune responses. The function and prevalence of these virulence factors among ExPEC strains are described below. While some factors have functions required for bacterial cell growth and survival, with an additional role in protection, others appear to be expressed solely to protect from immune responses. The bacterial cell envelope consists of a polysaccharide layer expressed on the external surface of the outer membrane, the outer membrane, the periplasm peptidoglycan layer, and the lipid inner membrane. Not surprisingly, considering that the host defenses are extracellular and rely on binding to and/or penetration of the bacteria, these bacterial resistance factors are either integral to the bacterial cell envelope, or are secreted externally. The bacterial factors that have been found to play a role in resistance to host defenses can be broadly categorized by their location and function. The next section provides a description of these factors, after which their mechanism of action is described in the section that follows.

### 4.1. Surface and Extracellular Matrix Polysaccharides

The predominant *E. coli* cell surface polysaccharides are the LPS O-antigen and capsular K-antigen. LPS is a major component of the outer membrane of Gram-negative bacteria and is essential for survival. It consists of three different structural components: lipid A, inserted in the outer membrane; a hydrophilic core polysaccharide chain; and O-antigen, a repeating hydrophilic oligosaccharide side chain that is directed to the exterior milieu and specific to the bacterial O-serotype [106]. The genes responsible for O-antigen biosynthesis are generally located in a highly polymorphic gene cluster known as the *rfb* locus [107]. The structure of the O-antigen is highly variable, mainly through a large diversity of sugar moieties and glycosyl bonds formed within and between the repeating units of the O-polysaccharides [108]. These differences are manifested through polymorphisms in the genes within and in some cases also outside of the *rfb* locus. A serotyping scheme was initiated for *E. coli* based on O- and flagellar antigens by Kauffman [109], and 185 different O-antigens are currently recognized in that scheme [37]. The large majority of ExPEC strains express the O-antigen [41], which give these bacteria their smooth colony morphology. O-antigen negative strains display a rough colony appearance and are rarely detected among human clinical ExPEC strains isolated from invasive or urinary infections.

K-antigens are found in many pathogenic *E. coli* isolates and are linked to the cell surface, where they form a polymeric capsular structure that extends up to hundreds of nanometers into the extracellular environment. Similarly to the O-antigen, the repeat unit structure of the K-antigens differs per serotype. To date, over 80 serologically distinct K-antigen polysaccharides have been described [110]. These can be classified into four different families (groups 1 to 4), based on assembly strategy and structural features [40]. Among ExPEC strains isolated from invasive infections, around 80% express the K-antigen (primarily from group 2 and 3) or have been identified to contain the biosynthesis locus [111,112].

Colanic acid is an extracellular matrix polysaccharide secreted by most *E. coli* strains, except those that produce group 1 capsules, and by other species of *Enterobacteriaeceae* [113]. It forms a slimy capsule surrounding the cell surface and protects the bacteria under hostile conditions such as osmotic shock, low temperatures, and desiccation [55,114]. Chemically, colanic acid is a negatively charged polymer of glucose, galactose, fucose, and glucuronic acid.

The enterobacterial common antigen (ECA) is an invariant carbohydrate antigen present in all LPS+ (smooth) members of the bacterial order Enterobacterales [115]. It is linked to the outer membrane via its phosphoglyceride portion (ECApg) and a version with shorter polysaccharides is present in a cyclic form in the periplasm (ECAcyc) [116].

### 4.2. Proteins Located in the Outer Membrane and Periplasm

Several proteins involved in serum resistance are embedded within the outer cell membrane of *E. coli*. These molecules have diverse structures and functions and may also act in concert to provide stability to the envelope structure. Murein lipoprotein (Lpp) is the most abundant protein in *E. coli*, according to the EcoCyc database [117], and this highly conserved lipoprotein is responsible for bacterial cell wall stabilization and integrity by joining the outer membrane and the peptidoglycan layers. About one third of the protein is covalently bound to the cell’s peptidoglycan layer while the remaining free protein spans the outer membrane and is surface-exposed [118]. Consequently, *E. coli lpp* mutants are more sensitive to the detergent SDS, suggesting decreased membrane integrity in the absence of Lpp [119].

Other lipoproteins that form part of the outer membrane are TraT, Iss and NlpI. Activation of the *traT* gene (encoded by ColV plasmids, such as the conjugative plasmid R6–5) causes a diminished capacity of plasmid-carrying bacteria to act as effective recipients in conjugation [120]. Despite being part of a mobile element, its prevalence among clinical ExPEC isolates is high: a recent meta-analysis of over 1800 UPEC strains in 8 countries found that over 75% of all strains contained TraT [121]. Iss, short for increased serum survival, is encoded by the *iss* gene located on ColV/BM plasmids, although variants of the gene are also found integrated in the *E. coli* genome. Overall, the genetic information for Iss is found in 70–90% of UPEC and neonatal meningitis, and in more than 90% of avian pathogenic *E. coli* (APEC) isolates for which this factor is a hallmark [122]. NlpI is a lipoprotein that interacts with peptidoglycan hydrolases to help maintain cell membrane integrity and is also highly prevalent among ExPEC strains [123].

Curli and outer membrane proteins (OMPs) are other conserved outer membrane structures. Curli are amyloid fimbriae that play an important role in bacterial adherence and enhance the formation of biofilms and are widely distributed in UPEC strains [124,125]. OMPs, of which OmpA is a predominant member that is highly conserved among *E. coli*, are beta barrel proteins that can have a variety of functions including a role in structural integrity of the outer membrane [126].

Several central carbohydrate metabolism enzymes present in the outer membrane were also identified as potential mediators of serum resistance, including acetate kinase AckA, fructose-bisphosphate aldolase FbaA, the fumarate reductase flavoprotein subunit FrdA, NADH-linked lactate dehydrogenase LDH, dihydrolipoyl dehydrogenase LpdA, pyruvate dehydrogenase Pdh, and phosphoenolpyruvate synthetase PpsA [127]. As carbohydrate metabolism is a fundamental process for bacteria, these proteins are essential among ExPEC, although outer membrane localization may be strain dependent.

As an additional defense layer just below the outer membrane, *E. coli* produces highly specific periplasmic lysozyme inhibitors such as Ivy (inhibitor of a vertebrate lysozyme), MliC (membrane-bound lysozyme inhibitor of c-type lysozyme), and PliG (periplasmic lysozyme inhibitor of g-type lysozyme) [128,129,130,131]. These proteins are conserved among *E. coli* and their sole function appears to be the defense against the lytic action of host lysozymes.

### 4.3. Secreted and Periplasmic Toxins and Proteases

Prominent soluble cytotoxins secreted by ExPEC are hemolysin (HlyA) and cytotoxic necrotizing factor 1 (CNF1). For both proteins a role in infection of the urinary tract has been established, although the reported prevalence of these factors among ExPEC strains is generally below 50%, with variation dependent on the patient population and specific lineage [132,133,134,135].

Other secreted toxins include members of the serine protease autotransporters of Enterobacteriaceae (SPATE) family, that are produced by both ExPEC and InPEC pathotypes [136,137]. SPATEs include class 1 cytotoxic and class 2 immunomodulatory proteases [138]. ExPEC strains often contain a combination of class 1 and 2 SPATEs, and there is a high degree of allelic variation and functional redundancy among these proteins. A prominent class I member is the secreted autotransporter toxin (Sat), a vacuolating toxin that interferes with F-actin function in a variety of epithelial cells [139,140]. It appears to be enriched among ExPEC strains, as it has been found in 68% of *E. coli* isolates from urine or blood of pyelonephritis patients but in only 14% of fecal *E. coli* strains from healthy women [141]. Among the ExPEC pathotypes, Sat is the most frequently observed SPATE, followed by class 2 SPATE Vat (vacuolating autotransporter protein) that induces intracellular vacuoles resulting in cytotoxic effects [142,143]. The plasmid-encoded class 1 SPATE protein EspP is widely distributed among Shiga toxin-producing *E. coli* but has also been found in some ExPEC isolates [144]. Pet, short for plasmid-encoded toxin, is a class 1 SPATE primarily associated with InPEC pathotypes, although one study reported a significant enrichment among bacteremic *E. coli* [145]. Among class 2 SPATEs, the temperature-sensitive hemagglutin (Tsh) is a prominent toxin present in about 50% of avian pathogenic *E. coli* [146] and in many UPEC strains [147]. Periplasmic protease Prc (also known as tail-specific protease Tsp) is another class 2 SPATE important for the integrity of the cell envelope [148]. Together with NlpI, Prc interacts with host peptidoglycan hydrolases [149], and the protease impacts ExPEC uropathogenesis by influencing regulation of flagellar expression and consequently the ability to cause UTI [150]. Class 2 SPATE protein involved in colonization (Pic) is a mucinase present in many enteroaggregative *E. coli* isolates [151] and in ExPEC isolates associated with pyelonephritis [147], where it is known as PicU [152].

## 5. Bacterial Mechanisms to Counteract Host Defense Strategies

**Figure 2:**
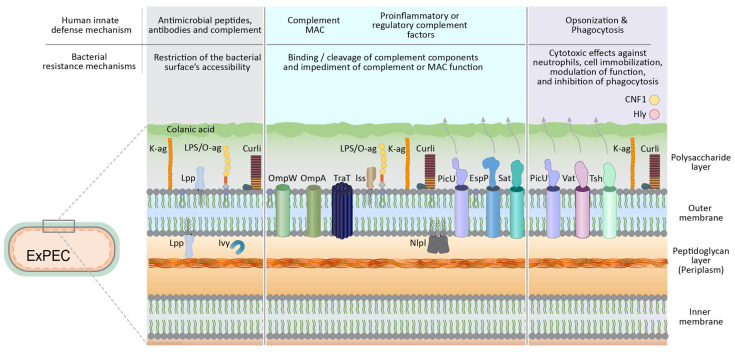
Major mechanisms and key factors involved in immune resistance by ExPEC, categorized in restriction of surface accessibility, impediment of the complement system and resistance to cell mediated killing. Human immune mechanisms are depicted at the top, and the corresponding bacterial counteractivity categories are shown with exemplary ExPEC molecules illustrated with their location on the bacterial cell. Molecular mimicry, a fourth bacterial resistance mechanism, is based on resemblance of bacterial capsule polysaccharides to host glycoproteins. Many of the molecules also work in concert to maintain cell envelope integrity in the face of immune attack.

As detailed in the previous sections, the human host can employ a variety of different immune mechanisms that work in concert to prevent infection with bacterial pathogens, while ExPEC can make use of an arsenal of virulence factors to withstand these host defense systems. The next sections discuss how these bacterial factors contribute to the three major strategies to avoid effective clearance by the host: (1), impediment of the function of the complement system; (2), restriction of access to the bacterial surface, thereby preventing effective recognition by AMPs, antibodies and PRMs; and (3), resistance against cell-mediated killing, as illustrated in Figure 2 and summarized in Table 1. Additionally, molecular mimicry by several ExPEC capsules, engaging inhibitory signaling pathways, and mechanisms to maintain bacterial cell integrity are also discussed in the context of bacterial resistance to host defense mechanisms.

### 5.1. Factors Impeding the Complement System

#### 5.1.1. Serum Resistance

Many components that are either part of the *E. coli* outer membrane or regulate its synthesis and maintenance play an important role in serum resistance. Genomic analyses have given insights into the specific bacterial factors involved in serum resistance. A comprehensive analysis was performed using transposon directed insertion-site sequencing in the O25b:H4-ST131 ExPEC strain EC958, a multi-drug resistant ExPEC strain isolated in 2005 from a patient suffering from a UTI [198], which revealed 56 genes involved in resisting the bactericidal activity of serum [119]. Over 20 of these were from three operons that are required for biosynthesis of LPS (including O-antigen and lipid A) and the enterobacterial common antigen (ECA). Genes encoding membrane proteins, lipoproteins, regulators, and hypothetical proteins constituted the remaining components of the serum resistome. Other factors were identified in a global transcriptome analysis of the O6:K2:H1 urosepsis. When ExPEC strain CFT073 was exposed to human serum, activation of three extra cytoplasmic stress response pathways, the Rcs and Cpx two-component systems and the alternative sigma factor σE, was detected [153]. While the Rcs two-component system improves serum survival by inducing colanic acid expression, Cpx and σE pathways regulate the structure and integrity of the outer membrane. Another global transcription profile using the O2:K1 ExPEC strain XM grown in serum under low oxygen conditions (2.5% O2, simulating existing conditions in the host tissue environment) confirmed genes involved in the biosynthesis of LPS, ECA, colanic acid, peptidoglycan, and exopolysaccharides to be upregulated in serum conditions compared to growth in Luria broth [199]. In this ExPEC isolate, transcription of genes of the capsule biosynthesis operon (*cps*) was also significantly increased in serum.

Several studies have shown that capsular polysaccharides can confer resistance to complement-mediated killing [200], although their effect on bacterial resistance against serum activity varies depending on the K-antigen structure. For example, while the K1, K2, K54 and related K96 capsular polysaccharides have been shown to contribute to *E. coli* survival in serum [45,154,155,201,202,203,204,205], this protection was not apparent in other capsule types, such as K15 [206], K27 [207], or the untyped class 2 capsule of ExPEC strain EC958 [119]. Additionally, *E. coli* expressing K1 capsular polysaccharide with elevated levels of O-acetylation displayed significantly higher blood CFUs in a mouse infection model, suppressed proinflammatory cytokine production and intracellularly inhibited vacuole fusion with lysosomes [208]. Finally, it was observed that group 4 capsule (also known as O-antigen capsule), a protective layer of polysaccharide made of the same strain-specific O-antigen as LPS, was required for serum survival in a septicemic O78 ExPEC isolate [209]. Periplasmic lipoprotein Lpp has also been found to play a key role in preventing complement-mediated bacterial lysis in vitro and complement-mediated clearance in vivo in the ExPEC model strain CFT073 [173]. At least part of this role is an indirect effect that can be attributed to its support in capsular polysaccharide assembly. Recently, a similar role was hypothesized for membrane-bound hemolysin, that was observed to mediate serum resistance likely through membrane stabilization by association with surface exposed polysaccharides [210]. Activation of the *traT* gene encoding the membrane lipoprotein TraT also resulted in increased resistance to serum, although this effect was independent from the bacterial capsule [179,180].

In line with the differential effects observed for capsular polysaccharides, serum resistance conveyed by colanic acid polysaccharides, which are structurally closely related to group 1 capsules, also appears to be isolate specific. Increased gene expression of the colanic acid biosynthesis operon was identified in transcriptome analyses of ExPEC strains that were exposed to serum, and colanic acid production protected ExPEC strains CFT073 (the prototypic urosepsis isolate) and RS218 (an NMEC isolate) from the bactericidal effects of the serum [153]. However, no serum-protective effect of colanic acid was observed in ExPEC strain CP9 (an O4:K54:H5 clinical blood isolate) [211], and its role in serum protection may therefore depend on regulatory networks of the specific isolate.

The O-antigen is another important contributor to serum resistance. Both the architecture and chain length of the O-antigen have been found to play a vital role in the protection of ExPEC against complement-mediated bactericidal killing [212]. The survival of three UPEC strains of differing O-antigen types (O1, O18, and O6) in the presence of serum was adversely affected by deletion of the O-antigen ligase gene *waaL*, a manipulation that prevents attachment of the O-antigen to the LPS core and causes a rough phenotype [213]. Furthermore, in the serotype O75 expressing UPEC strain GR-12, mutants in *rfbD* (involved in TDP-rhamnose synthesis), *rfbKM* (required for GDP-mannose synthesis), and *rol* (regulated O-antigen chain length, also known as *wzz*) displayed a rough phenotype, a partial O-antigen subunit, and random pattern of O-antigen chain length phenotypes, respectively. These mutants were all more sensitive to complement-mediated lysis than the wildtype parental strain [212].

#### 5.1.2. Factor H Binding

Many Gram-negative pathogens escape complement killing by binding of regulators of complement activation, including factor H, the negative regulator of the AP that destabilizes the C3 convertase (Figure 1) [214]. For example, both capsular sialic acid and OmpW protect *E. coli* bacterial cell surfaces from attack by the AP by recruiting and binding factor H [156,157,158,178,215]. Binding of polyanions like heparin, sulphated glycosaminoglycans, and polysialic acid may also increase the interaction of factor H with C3b and lead to stronger downregulation of AP complement activation [159].

In addition, an arsenal of seven central carbohydrate metabolism enzymes was shown to contribute to binding of factor H in the ExPEC strain RS218 [127]. Some of these (FbaA, Pdh, and LDH) have been suggested to be moonlighting central metabolism proteins that have additional functions in other pathogens, including factor H binding (FbaA in *S. pneumoniae* and *N. meningitidis* [216,217]). While these enzymes are present in the *E. coli* outer membrane, no functional studies have yet elucidated how exactly they recruit factor H to the bacterial surface and how a possible defect in their factor H-binding functionality would affect ExPEC survival in human serum.

#### 5.1.3. AP/CP Disruption

In addition to harnessing factor H, resistance to complement-mediated killing can occur by binding or degradation of other factors in the complement pathways. C4b-binding protein (C4bp), a complement regulating protein that prevents formation of C3 convertase in the CP to avoid uncontrolled activation of this pathway (Figure 1), is recruited by various *E. coli* resistance factors including OmpA and Nlpl [175,176]. In in vivo murine models, the curli of wild type *E. coli* strains Nissle 1917 (a semi-rough O6:K5:H1 commensal isolate) and MC4100 (a rough laboratory strain) protected the bacteria from complement-mediated killing by the classical pathway [167]. Although the exact mechanism was not elucidated, potential binding of curli to C4bp was hypothesized based on the finding that the structurally related Alzheimer’s associated protein amyloid beta binds C4bp [218].

Other molecules disrupt complement factors via enzymatic activity. For some SPATEs, protease activity on complement factors has been shown. Sat, the most prominent SPATE, was found to exert proteolytic activity on C2, C3, C3b, C4, C4b, C5, C6, C7, C8, and C9 [186]. Pic is a mucinase that can cleave complement proteins C2, C3, C3b, C4, and C4b [191], and deletion of *pic* resulted in the attenuation of virulence in a mouse model and a lack of survival in the bloodstream [219]. The plasmid-encoded *E. coli* secreted serine protease (EspP) acts on complement factors C3, C3b, and C5 [144,188,189]. Pet was found to cleave C3, C5 and C9 components, inhibited the natural formation of C9 polymers, and human serum pretreated with Pet conferred survival on the *E. coli* laboratory strain DH5α [190]. The periplasmic protease Prc plays a role in the bacterial evasion of complement attack mediated by the CP, although proteolytic activity on complement components could not be established [193].

#### 5.1.4. MAC Inactivation

The MAC causes lysis of the bacteria by forming holes in the pathogen’s cell envelope (Figure 1). Some bacterial factors act to prevent MAC formation or action. TraT inhibits MAC activity [171], likely by preventing the formation of the C5b6 complex [181]. The O-antigen has been demonstrated to impair polymerization of C9 thus preventing formation and integration of the complete MAC into the bacterial cell wall [70]. LPS with a shorter or no O-antigen cannot resist this insertion and the *E. coli* membrane becomes readily available for MAC-mediated lysis of the bacteria [57,162,220].

Iss may play a role in the formation of functional complexes in the junctions between the inner and outer membranes and help in preventing an attack by the MAC [171]. In the development of septicemia, the presence of Iss in pathogenic *E. coli* was highly linked with complement resistance and mortality [172]. Loss of the *iss* gene lowered group 4 capsule (O-antigen) production in a septicemic strain of *E. coli* O78, caused serum sensitivity, and changed the pattern of complement proteins that bound to the bacteria [172]. In addition, the curli of wild type *E. coli* strains Nissle 1917 (a known probiotic) and MC4100 (a laboratory strain) were found to mediate resistance to classical complement pathway mediated killing in in vivo murine models, possibly by providing a physical barrier to MAC integration [167].

### 5.2. Factors Restricting Access to the Bacterial Surface, Thereby Preventing Effective Recognition by Antibodies, AMPs, and Complement

*E. coli* expresses several factors that prevent the action of antibodies, AMPs, and complement factors. This resistance is imparted through inhibition of binding, or deactivation. While the specific mechanisms of protection from immune responses by biofilm are beyond the scope of this review, it is worth noting that the majority of UPEC strains produce biofilms [221], particularly those causing recurrent UTIs. Curli is a main component of *E. coli* biofilms, the formation of which hampers the effectiveness of innate immune responses against Gram-negative bacteria. The biofilm presents a physical barrier that restricts access to the bacterial surface [168,169].

There are several ways by which antibodies can promote bacterial clearance, but all these mechanisms rely on antibody binding to the bacterial surface. The highly glycosylated envelope already provides some protection from antibody responses because, in general, antibodies produced against polysaccharides are of lower affinity, compared with the T-cell dependent production of higher affinity antibodies against protein antigens [222].

Within the LPS, the surface-exposed O-antigen is a prime target for the attachment of host antibodies. This observation has been utilized for the development of *E. coli* vaccines. O-antigen-based conjugate vaccines were found to induce functional opsonophagocytic antibodies and have shown promising results in mice [223,224] and humans [225,226,227,228,229], although a recent phase 3 clinical trial was discontinued due to lack of sufficient vaccine efficacy [230]. O-antigen-targeting high-affinity monoclonal antibodies have been shown to provide protection against *E. coli* in animal models [231,232]. However, in response to colonization or infection with a bacterial pathogen, some human hosts produce high amounts of antibodies specific to O-antigen which may protect the bacteria from complement-mediated lysis instead of targeting the invader for destruction. This was described for about 25% of patients suffering from UTI caused by *E. coli* or *P. aeruginosa* [163,164]. Existence of such “cloaking antibodies” of various types (IgG, IgA, IgM) with different targets (most often LPS, i.e., O-antigen) have also been described in *S. enterica, N. meningitidis* and *Burkholderia* [233,234], although their overall impact on the course of bacterial infections remains controversial.

In addition to protection from antibody responses, several bacterial factors also exhibit resistance to AMPs. Notably, *E. coli* can modify their surface LPS with the help of LPS-decorating enzymes such as phosphoethanolamine transferase EtpA and glycosyltransferase ArnT by adding cationic compounds to the lipid A moiety that repel AMP binding [165]. Furthermore, *E. coli* surface LPS can bind lysozyme and inhibit its enzymatic action, and O-antigen is crucial for this protection in ExPEC isolates [131]. Lysozyme is also the target of highly specific periplasmic lysozyme inhibitors such as Ivy, MliC, and PliG [128,129,130,131]. Further studies have shown resistance of *E. coli* to other AMPs. For instance, the O-antigen contributed to protection of Enteroaggregative *E. coli* (EPEC) from the human α defensin HD-5 [235], and curli demonstrated resistance against the human cathelicidin LL-37 in an UPEC isolate [170].

The complexity of the role of bacterial surface molecules in cell integrity, cell function, and cell defense is illustrated by the cell surface molecule Lpp. Lpp is needed for cell wall stability and contributes to the defense against complement- and neutrophil-mediated killing. However, Lpp may also be a target of RNAse 7, the most abundant innate defense peptide in the human urinary tract with antibacterial properties against uropathogens including *E. coli* [236,237]. This AMP induces local membrane destabilization [238,239], possibly via interactions with Lpp [240].

### 5.3. Factors Resisting Neutrophil- or Cell-Mediated Killing

Neutrophils are usually the first line of cellular defense against bacterial infection, although other immune cells, such as macrophages, are also recruited to contain infection. Bacterial strategies to avoid cell-mediated killing involve cell immobilization, modulation of function, inhibition of phagocytosis, and a cytotoxic counterattack.

The curli-enriched biofilm layer produced by *E. coli* can function to immobilize neutrophils and render the bacteria inaccessible to cell-mediated immunity [168,169]. In addition, SPATEs can act on the neutrophil surface glycoproteins to impede cellular functions. For example, in addition to its effect on complement proteins, Pic digests O-linked glycoproteins such as CD43 and CD45 commonly found on neutrophil surfaces [192,241] and thereby modulates their function. Furthermore, Tsh was also reported to target a variety of glycoproteins on the surface of neutrophils [192], and recently CD43 was identified as a target for Vat and Tsh homologues in a porcine ExPEC strain [194].

Another protection mechanism of ExPEC against a neutrophil-mediated immune response of the host is provided by cytotoxins that impede the function of these immune cells. Hemolysin (HlyA) is a prominent exotoxin that is expressed and secreted by ExPEC and induces the formation of membrane pores in host cells [242]. The production of hemolysin significantly correlates with a higher extent of serum resistance in O4, O6, O18, and O75 *E. coli* isolates [187]. In in vitro models, the clinical ExPEC isolate CR9 induced either cell apoptosis or necrosis/lysis when neutrophils were exposed to 10^5^ or 10^6^ CFU of bacteria, respectively. This phenotype was curtailed when a *hlyA*- mutant of the strain was used [243]. Hemolysin therefore contributes to premature neutrophil death and consequently reduces neutrophil contact for ExPEC, increasing the bacteria’s odds of survival. The exotoxin CNF1 has also been implicated in the capacity of UPEC to resist killing by neutrophils. In vitro experiments showed that CNF1-positive *E. coli* strain CP9 was better able to resist killing by fresh human neutrophils than were isogenic CNF1 knock-out bacteria [183]. Additionally, the toxin’s production increased survival of UPEC strains in association with isolated human neutrophils [184] and CNF1 treatment of neutrophils decreased their ability to phagocytize bacteria [185].

The cell-surface linked capsular polysaccharides are considered crucial for evasion of neutrophil-mediated bacterial killing [244]. It has been shown that capsular polysaccharides including K1 can confer resistance to phagocytosis, by inhibition of complement activation and C3b or C3d binding to the bacteria [45,200,245], which also prevents direct complement-mediated killing mechanisms. Moreover, capsule types K1 and K5, but not K2ab and KG2-1, protected ExPEC strains from recognition by murine Kupffer macrophages in the liver in a murine sepsis model [160].

Using a different mode of action to combat neutrophils, Lpp may inhibit the production of reactive oxygen species by these cells, possibly indirectly by downregulating expression of bacterial flagellin [174].

### 5.4. Factors Involved in Molecular Mimicry and Immune Cell Inhibition

Several *E. coli* capsular polysaccharides such as the K1 antigen (α-(2-8)-linked polysialic acid), K4 (a substituted chondroitin backbone), and K5 (an N-acetylheparosan backbone) resemble host glycoproteins [110]. For instance, the polysialic acid structure of the K1 capsule is abundantly present on neuronal cells in the human brain. This molecular mimicry may prevent recognition by pathogen-specific antibodies and PRMs. Moreover, extracellular sialic acid-containing glycans are also recognized by sialic acid-binding immunoglobulin-like lectins (SIGLECs, reviewed in [246]). SIGLECs are receptors on immune cells that often contain intracellular immunoreceptor tyrosine-based inhibition (ITIM) motifs. Binding of bacterial sialic acid-containing glycans to these receptors can thus counteract tyrosine-based activating (ITAM) motifs from activating receptors [247] and thereby decrease the activation of the immune cells. Of note, many other important Gram-negative and Gram-positive pathogens, including group B streptococci, *H. influenzae*, *Pseudomonas aeruginosa*, *Campylobacter jejuni*, and *N. meningitidis* serogroups B, C, Y, and W, also contain sialylated glycans on their cell surface [248,249], likely contributing to resistance of these bacteria to the human immune system.

### 5.5. Factors Supporting Membrane Integrity, the Interplay of Envelope Components in Resistance and Regulatory Networks

The integrity of the bacterial cell envelope plays a vital role in the pathogen’s survival, and the intricate interplay of all cell envelope components is key to the success of bacteria as human pathogens. While individual envelope components play their own role in protection from immune responses, the combination of the different factors in maintaining cell integrity contributes to resistance to immune attack. For example, the role of capsular polysaccharides in immune evasion can vary per strain, as shown in a study exploring the effects of capsule deletion on one K2 and two K1 UPEC strains of different O-antigen types. Capsule deletion resulted in faster killing in whole blood in the K2:O6 and K1:O1 strains, whereas killing in serum was only increased in the K2:O6 and K1:O18 capsule mutants [213]. Similarly, double mutants that were lacking both the K5 capsule and the O-antigen were more sensitive to serum-killing than single mutants devoid of either [212]. The role of capsule in resistance to phagocytosis or direct killing in serum may therefore be linked to the O-antigen or be specific to each capsule architecture.

The O-antigen also interacts with ECA: ECA can be attached to the LPS core in bacteria where O-antigen production is inhibited (rough strains), where it is immunogenic, or can be linked to the diacylglycerol of peptidoglycan in smooth strains, where it is non-immunogenic [250]. Its exact function in resistance against the host immune response is surprisingly difficult to ascertain—but the cyclic, periplasmic form of ECA contributes greatly to the outer membrane permeability barrier [251].

Multiple immune resistance factors are associated with envelope integrity. Nlpl interacts with several peptidoglycan endopeptidases [252], directly affecting cell envelope stability. *E. coli lpp* mutants are more sensitive to SDS, suggesting decreased membrane integrity in the absence of Lpp [198]. Furthermore, point mutations in the *traT* coding sequence dramatically increase permeability of the outer membrane [182]. Transcriptional regulators such as Rcs, Cpx, and σE are activated in response to cell envelope stress in an effort to maintain membrane integrity [253].

Environmental cues can also trigger regulation of expression of virulence factors associated with serum resistance via quorum sensing (QS). This cell-to-cell communication network uses autoinducer (AI) molecules that initiate a signal transduction cascade when threshold concentrations are reached. *E. coli* produces several AI that play a role in bacterial survival and colonization in diverse niches [252]. For ExPEC, biofilm formation is a prominent feature regulated by QS, via AI such as autoinducer 2 [254].

## 6. Conclusions

The human host uses several defense strategies to limit the overgrowth of commensal bacteria and prevent pathogenic infections. The primary defense is provided by the innate immune response, which includes a precisely regulated complement system with its final membrane attack complex, and soluble antimicrobial peptides and proteins. The host also employs opsonophagocytic killing pathways, some of which depend on activation of the complement system or include antibody-mediated mechanisms. However, there is a symbiotic relationship between the human host and bacteria, and bacteria display a degree of resistance to these immune responses to allow for their long-term survival. This balance is lost when *E. coli* (and ExPEC in particular) develops a combination of resistance mechanisms that enable the bacteria to thrive in the host environment by significantly diminishing or incapacitating one or more of these host protection mechanisms. This review limits its focus to the *E. coli* factors with specifically described roles in resistance against human immune responses. These factors, which are present in different combinations in different pathogenic *E. coli* isolates, contribute to one or more bacterial immune resistance categories, including: binding of and/or prevention of function of complement components and regulators, restriction of the bacterial surface’s accessibility, cytotoxic effects against neutrophils, and molecular mimicry. It is likely that some of these components act in concert to exert their protective effect, for example, by together stabilizing the bacterial cell membrane when it comes under attack. The notable absence of *E. coli* as a breakthrough organism in human hosts with complement, antibody, or antimicrobial peptide deficiencies contributes to the concept that there is redundancy in the modes of action of immune protection, and also that a multifaceted immune response is required to maintain the status quo. Mechanisms such as cell-mediated killing, although also partly dependent on specific complement factors or antibodies, are of significance in the human defense arsenal against ExPEC. This is exemplified by the increased susceptibility towards *E. coli* infections seen in individuals suffering from neutrophil deficiencies [78] and increasing incidence rates of invasive *E. coli* disease including bacteremia and sepsis in adults of advanced age [10], where neutrophil function is declining.

The capsule and O-antigen as well as its arsenal of cytotoxins and SPATEs have been shown to be of particular importance in the ability of pathogenic *E. coli* to circumvent direct complement-mediated killing and phagocytosis. However, vaccines targeting the O-antigen have been found to elicit specific antibodies to this key virulence factor that induce opsonophagocytic killing. Although these high-affinity functional antibodies might be expected to shift the delicate balance and overcome the complex bacterial defense mechanisms, a recent clinical trial showing lack of efficacy for an O-antigen-targeting vaccine illustrates the complexity of these interactions [230]. Our understanding of the exact mechanisms of how ExPEC counteracts the human innate immune system has not advanced sufficiently to fully delineate the entire arsenal of components that play a role in this pathogen’s resistance ultimately leading to a successful strategy to prevent ExPEC infections. Given the increasing development of antimicrobial resistance (AMR) by bacterial pathogens, resulting in increased morbidity and mortality from bacterial infections with *E. coli* as a leading pathogen [255,256,257], research in this field is of particular importance. Moreover, by synergistic mechanisms AMR may lead to increased resistance to host immune mechanisms [258], which could further complicate the development of effective prophylactic or treatment options.

Several different antibacterial strategies against ExPEC are currently in development, that could form an alternative for or complement traditional antibiotics. These efforts include a broad range of compounds targeting various bacterial factors, among which whole-cell, subunit or conjugate vaccines [259], monoclonal antibodies, antimicrobial peptides, small molecules, nanoparticles and bacteriophages [260]. Future studies reporting on these approaches will inform us on the most promising avenues to follow and give insights into immune biomarkers that signal a protective response. More research into the underlying causes of the observed increased prevalence of systemic ExPEC infections in human subpopulations such as older adults will hopefully elucidate additional aspects of the complicated network of host–pathogen interactions and reveal molecular targets that can be used to counter the detrimental effects of increasing immune-senescence. Development of antibacterial therapies will be aided by a more thorough understanding of how innate and adaptive immune responses can be directed to overcome bacterial resistance mechanisms.

## Figures and Tables

**Table 1:** Prominent ExPEC factors that contribute to resistance against the human innate immune response by different mechanisms, categorized by their cellular function and/or location.

Factor	Mechanism(s) of Resistance				References
	Impediment of Complement System	Restriction of Access to Bacterial Surface	Resistance Against Cell-Mediated Killing	Other	
**Surface and extracellular matrix polysaccharides**					
Colanic acid (M-antigen)	Serum resistance	Prevents access to bacterial cell envelope			[153]
ECA	Serum resistance			Capsule integrity	[119]
K capsule (K-antigen)	Serum resistance Downregulates complement activation Increases interaction factor H (regulator of AP) with C3b	Provides a steric barrier to prevent deposition of complement factors	Resistance to phagocytosis Downregulates opsonization	Possible molecular mimicry to prevent pathogen recognition Bind to SIGLECs, negative regulation of immune cells Capsule integrity	[154,155,156,157,158,159,160,161]
LPS and O-antigen	Serum resistance Impairs C9 polymerization and MAC insertion into the membrane	Recruits cloaking antibodies Protects from AMP		Cell envelope integrity	[70,131,162,163,164,165,166]
**Outer membrane and periplasmic proteins**					
Carbohydrate metabolism enzymes AckA, FbaA, FrdA, LDH, LpdA, Pdh, PpsA	Bind factor H (regulator of AP)				[127]
Curli	Binds C1q and inhibits CP Barrier to MAC integration	Physical barrier to prevent access to bacterial surface AMP resistance (cathelicidin LL-37)	Immobilize neutrophils		[167,168,169,170]
Iss	Serum resistance May inhibit MAC insertion	Governs production of O-antigen			[171,172]
Lpp	Prevents complement-mediated lysis and clearance		Inhibits reactive oxygen species in neutrophils	Cell envelope integrity	[173,174]
Nlpl	Facilitates interaction between C4bp and OmpA to inhibit CP activation			Cell envelope integrity	[119,123,175]
OmpA	Binds C4bp and inhibits complement activation via the CP				[176,177]
OmpW	Binds factor H (regulator of AP)				[178]
Periplasmic lysozyme inhibitors Ivy, MliC, PliG		Inhibit the AMP lysozyme			[128,129,130]
TraT	Serum resistance Inhibits the formation of the C5b6 complex			Cell envelope integrity	[171,179,180,181,182]
**Toxins and proteases**					
CNF1 toxin			Decreases the ability of neutrophils to phagocytize bacteria		[183,184,185]
HlyA toxin	Serum resistance		Formation of membrane pores causes neutrophil apoptosis or lysis		[186,187]
**SPATE class I cytotoxins**					
EspP	Protease acts on complement factors C3, C3b, and C5				[144,188,189]
Pet	Cleavage of complement proteins C3, C5 and C9				[190]
Sat	Cleavage of complement proteins				[186]
**SPATE class II immunomodulators**					
Pic or PicU	Cleavage of complement proteins C2, C3, C3b, C4, and C4b		Digests O-linked glycoproteins on the surface of neutrophils and lymphocytes		[191,192]
Prc (Tsp)	Evasion of CP				[193]
Tsh			Targets surface glycoproteins on neutrophils		[192,194]
Vat			Targets surface glycoproteins on neutrophils		[194]
**Transcriptional regulators**					
Cpx, σ^E^				Capsule integrity in response to envelope stress	[195,196]
Rcs				Induces production of colanic acid in response to envelope stress	[197]

AckA, acetate kinase; AMP, antimicrobial peptide; AP, alternative complement pathway; C4bp, C4b-binding protein; CNF1, cytotoxic necrotizing factor 1; CP, classical complement pathway; Cpx, conjugative pilus expression; ECA, enterobacterial common antigen; EspP, extracellular serine protease; FbaA, fructose-bisphosphate aldolase class 2; FrdA, fumarate reductase flavoprotein; HlyA, hemolysin; Iss, increased serum survival protein; Ivy, inhibitor of vertebrate lysozyme; LDH, Lactate dehydrogenase; LpdA, lipoamide dehydrogenase; Lpp, murein lipoprotein; MAC, membrane attack complex; MliC, membrane-bound lysozyme inhibitor of C-type lysozyme; NlpI, outer membrane lipoprotein; Omp, outer membrane protein; Pdh, pyruvate dehydrogenase; Pet, plasmid-encoded toxin; Pic, protein involved in intestinal colonization; PliG, Inhibitor of g-type lysozyme; PpsA, phosphoenolpyruvate synthase; Prc, periplasmic protease; Rcs, regulator of capsule synthesis; Sat, secreted autotransporter toxin; TraT, DNA transfer protein; Tsh, thermostable hemagglutinin; Vat, vacuolating autotransporter toxin.

## Data Availability

This manuscript is a review and no new data were generated or analyzed. All data and findings discussed are from previously published sources, which are cited in the text and listed in the References.
